# Investigating Financial Incentives for Maternal Health: An Introduction

**Published:** 2013-12

**Authors:** Mary Ellen Stanton, Elizabeth S. Higgs, Marge Koblinsky

**Affiliations:** ^1^United States Agency for International Development, Washington, DC, USA; ^2^National Institute of Allergy and Infectious Diseases, Bethesda, MD, USA

**Keywords:** Health services research: Maternal health services, Maternal welfare/economics, Pregnancy, Programme evaluation

## Abstract

Projection of current trends in maternal and neonatal mortality reduction shows that many countries will fall short of the UN Millennium Development Goal 4 and 5. Underutilization of maternal health services contributes to this poor progress toward reducing maternal and neonatal morbidity and mortality. Moreover, the quality of services continues to lag in many countries, with a negative effect on the health of women and their babies, including deterring women from seeking care. To enhance the use and provision of quality maternal care, countries and donors are increasingly using financial incentives. This paper introduces the JHPN Supplement, in which each paper reviews the evidence of the effectiveness of a specific financial incentive instrument with the aim of improving the use and quality of maternal healthcare and impact. The US Agency for International Development and the US National Institutes of Health convened a US Government Evidence Summit on Enhancing Provision and Use of Maternal Health Services through Financial Incentives on 24-25 April 2012 in Washington, DC. The Summit brought together leading global experts in finance, maternal health, and health systems from governments, academia, development organizations, and foundations to assess the evidence on whether financial incentives significantly and substantially increase provision, use and quality of maternal health services, and the contextual factors that impact the effectiveness of these incentives. Evidence review teams evaluated the multidisciplinary evidence of various financial mechanisms, including supply-side incentives (e.g. performance-based financing, user fees, and various insurance mechanisms) and demand-side incentives (e.g. conditional cash transfers, vouchers, user fee exemptions, and subsidies for care-seeking). At the Summit, the teams presented a synthesis of evidence and initial recommendations on practice, policy, and research for discussion. The Summit enabled structured feedback on recommendations which the teams included in their final papers appearing in this Supplement. Papers in this Supplement review the evidence for a specific financial incentive mechanism (e.g. pay for performance, conditional cash transfer) to improve the use and quality of maternal healthcare and makes recommendations for programmes and future research. While data on programmes using financial incentives for improved use and indications of the quality of maternal health services support specific conclusions and recommendations, including those for future research, data linking the use of financial incentives with improved health outcomes are minimal.

## INTRODUCTION

Globally, the UN estimates show that maternal mortality has fallen 47% between 1990 and 2010 (1). Even so, many countries are still not on track to achieve the UN Millennium Development Goal 5 (MDG 5) of 75% reduction in maternal mortality by 2015. Close to 290,000 women continue to die annually due to complications from pregnancy and childbirth (1). The goal to reduce maternal mortality remains challenging. In part, the toll of preventable maternal deaths represents persistent and heartbreaking inequities between the poor and the rich as well as between the uneducated and the educated. Over a lifetime, women are approximately 100 times more likely to die as a result of pregnancy in sub-Saharan Africa than in developed regions of the world. More than half of all maternal deaths occur in only eight countries, and 80% of all deaths occur in 22 countries (1).

The estimates of maternal death only capture a portion of the overall burden of maternal complications and disability on women and their families, communities, and societies. A recent study in Bangladesh found that, for every maternal death, there are 40 women who suffer obstetric complications, ranging from less severe to severe, and over 160 women live with postpartum morbidities and disabilities (2). This toll is much higher than previously thought (3). Postpartum morbidities and disabilities include such physical consequences as incontinence, uterine prolapse, obstetric fistula, anaemia, and hypertension. Following intrapartum complications and, sometimes, perinatal death, there can also be psychological consequences, such as postpartum depression and profound social consequences, including emotional, physical and sexual violence (2). Furthermore, the cost of maternal complications can result in debt that may compound emotional consequences and result in not seeking needed additional care.

Maternal death also has serious consequences for the survival of children. In Bangladesh, “infant mortality is approximately eight times higher for those infants whose mothers died than if the mother survived” (4). In addition, “the cumulative probability of survival to age 10 years was 24% in children whose mothers died before their 10th birthday compared to 89% in those whose mothers remained alive” (4).

Women in low- and middle-income countries (LMICs) with a high burden of maternal death often underutilize family planning and maternity services that facilitate healthy childbirths, save maternal and newborn lives, and ensure a healthy start for their young children. To address the lack of utilization and quality of maternal health services, many countries have recently used financial incentives (FIs). Evidence of the effectiveness of FIs for improved maternal healthcare was reviewed at the US Government Evidence Summit on Enhancing Provision and Use of Maternal Health Services through Financial Incentives convened by the US Agency for International Development and the US National Institutes of Health on 24-25 April 2012 in Washington, DC. The papers in this JHPN Supplement provide an overview of each of the financial incentives, along with conclusions and recommendations based on the literature reviewed and presentations made at the Evidence Summit.

### Inadequate use of lifesaving services

Women's use of healthcare services that potentially reduce the risk of death due to pregnancy is very low in many parts of the world. For example, contraceptive prevalence is 24% in Africa and 58% in South-East Asia compared to the 71-80% coverage in countries with high and upper mid-level income (5) where maternal mortality is demonstrably lower. Despite recent increases in facility-based delivery (6), 48% of women in Africa and 59% of women in South-East Asia still give birth without the presence of a birth attendant (5) who can recognize complications and provide stabilization and referral for emergency lifesaving care. Women who experience serious complications, such as prolonged/obstructed labour or severe pre-eclampsia/eclampsia, may require caesarean sections for health or survival of themselves or their newborns. While the optimal level of caesarean section is a matter of controversy, the 4% rate in Africa and the 9% rate in South-East Asia (5) highlight the need for better access to surgery and other specialized lifesaving services. Poverty, geographical location, and lack of specialized care, along with lack of recognition of the need for such care, all contribute to such low use-rates in LMICs. These coverage indicators are indicative of access and utilization of care but provide no information about the quality of care which has proven more difficult to capture.

### Barriers to the use and provision of quality maternity care

To focus the Evidence Summit on the critical barriers women face, which could prevent them from availing of services that could help them plan for and promote healthy pregnancy and birth, USAID carried out a scoping exercise in 2011. In this exercise, maternal health experts and teams from various international health organizations identified the following categories of barriers: family and community factors, health system constraints, poor quality of services, and governance issues leading to poor accountability at higher levels. Within these categories, important elements contributing to the low use and quality of services were identified (Figure).

Lack of knowledge and awareness among women, families, and decision-makers may contribute to delays in recognizing the initiation of labour and the need for skilled birthing care, especially urgent in the event of an obstetric complication. Distance to facilities, poor road conditions, seasonal problems, and lack of transportation can lead to delays in the decision to travel to service facilities. The costs associated with transport, care, and medications as well as the opportunity costs of family members travelling with the woman may also affect decisions to seek care. Furthermore, village or facility ‘gatekeepers’, through ignorance or corruption, may delay or prevent women in labour or with complications from accessing the services that would save their lives. Fundamentally, the lack of agency that denies women the ability to make informed decisions independently can often lead to non-use of maternity services or delays in accessing care.

**Figure.**
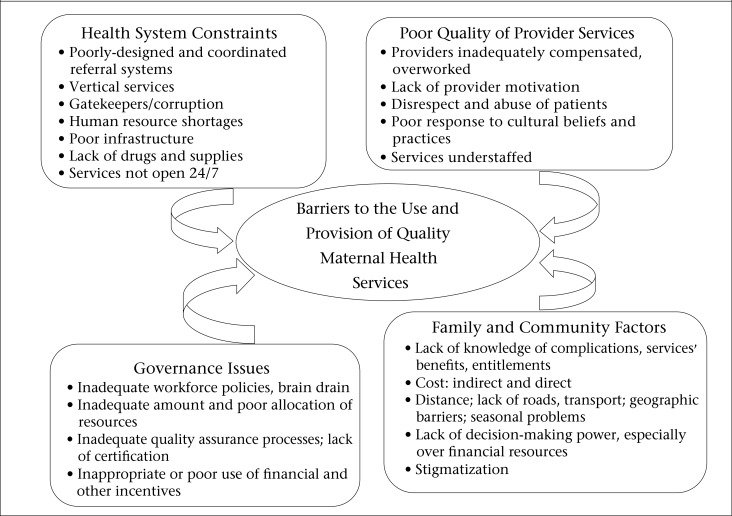
Elements contributing to the low use and quality of services

On the supply side, there are also many factors that negatively effect provision of quality care. For example, poorly-designed and coordinated referral systems can prevent or slow access to emergency care. Inadequately-integrated and coordinated vertical programmes can also result in missed opportunities to provide essential maternal care. Services that should be available on a 24-hour basis may not be open or staffed at night or on weekends. Inferior medical infrastructure, including lack of water or electricity and essential drugs and equipment, may also affect the capacity to provide even the most basic level of care.

Moreover, inadequate, poorly-trained and/or disempowered staff may provide substandard care. Dysfunctional incentives in the health system, such as low salary structures that do not vary based on performance, inadequate mechanisms to support, supervise, and hold health workers accountable, may result in low levels of motivation, absenteeism, and lack of innovation and quality. Healthcare providers may, in some cases, neglect or verbally or physically abuse women in outpatient clinics and even in childbirth in health facilities, thus discouraging the use of services and/or providing low quality of care that contributes to loss of life.

At the policy level, poor political commitment and weak governance can lead to low standards, inadequate budget, lack of supervision, graft, and services that are simply not accountable to the women and communities they are supposed to serve.

### Rationale for choice of financial incentives as key to the use and provision of improved maternal healthcare

Among the many barriers that contribute to the underutilization of lifesaving services, financial barriers are significant, especially on the demand side. In 20 countries, with more than one recent Demographic and Health Survey (DHS), half (52%) of women surveyed, on average, identified lack of money as the biggest problem to using health services (7). Financial incentives, either in the form of transfer of funds or reduction or elimination of direct customary charges that encourage a behaviour or action by the woman and family, have the potential to significantly reduce economic barriers posed by direct and informal fees as well as costs relating to transport, meals, lodging of accompanying family members, and other related expenses. On the supply side, although overall funding for the health sector is certainly needed, FIs can address such systemic disincentives as poor provider motivation, insufficient empowerment, and lack of accountability for results.

An important criterion for choice of inquiry through the Summit process was the importance of a barrier that could be improved through programmatic guidance based on available evidence. The importance of FIs and their evaluation is supported by the World Bank, its extensive experience in result-based financing for health (8), and by many health economists (9). The criteria are more fully described within this collection in Evidence Acquisition and Evaluation for Evidence Summit on Enhancing Provision and Use of Maternal Health Services through Financial Incentives by Higgs *et al*. in this Supplement.

Many countries and donors have turned to financial incentives in the last decade as a solution to underutilization and poor quality of maternal services. Various financial instruments, including vouchers, performance-based incentives, conditional cash transfers, and others, are being implemented in a variety of settings with the broad goal of reducing maternal mortality and morbidity by enhancing the use and quality of maternal health services. The motivation for the Summit was the global demand to demonstrate linkages between financial incentive instruments and health outcomes.

## MATERIALS AND METHODS

### Process of Evidence Summit

A systematic review of relevant English language publications since 1990 was undertaken, utilizing eight standard databases. There was a subsequent call for relevant additional papers from Expert Review Team members. In all, 139 references were provided to the Expert Review Teams to serve as the basis for a summary of findings and recommendations. The methodology for the literature review is described in the article by Higgs *et al*.in this Supplement.

Prior to the Summit, three evidence review teams comprised 56 experts in finance, maternal health, and health systems, reviewed and analyzed the evidence of various financial mechanisms, including: (i) supply-side mechanisms, such as performance-based financing, user fees, and various insurance mechanisms; (ii) conditional cash transfers in which programmes condition cash payments to poor households on the use of maternal health services; and (iii) other demand-side incentives, such as vouchers for the use of services and of insecticide-treated bednets, user fee exemptions, and subsidies for such items as food and transport to the clinic. The analyses focused on two key questions to assess the effect of financial incentives on the provision and use of maternal health services:

1. What financial incentives, if any, are linked positively or negatively to maternal and neonatal health outcomes, the provision and use of maternal health services, or to care-seeking behaviour by women?
2. What are the contextual factors that impact the effectiveness of these financial incentives?

At the Summit, the experts presented a synthesis of the evidence and analyses to a wider audience with the goal of formulating recommendations on practice, policy, and research. The Summit enabled structured feedback and dialogue on the conclusions and recommendations of the reviews. The Evidence Summit also explored cross-cutting issues and challenges that Summit participants advised the global community to address in future programming, research, and investment decisions.

### Cross-cutting issues of context, design, and implementation

The success of programmes using financial incentives for maternity care depends, in part, on the context within which they are implemented and the design of the FI. To explore fully how the context and design of the incentive programme influence outcomes, key questions were identified during the initial evidence review and then during the Summit to identify factors that could influence FI programme results (Table).

Evidence review teams reviewed the available literature with these questions in mind, and Summit participants shared their experience and debated some of these issues.

**Table.** Questions to identify contextual variables linked with results of financial incentives for the provision and use of maternity services

What contextual factors (e.g. proportion of the population that is poor, geography, urban/rural mix, state of the health system infrastructure) influence the choice, implementation and effectiveness of a specific incentive instrument?
What evidence is there for selecting and setting incentives correctly, including the type of incentive, and the ‘unit price’ of the incentive—both as a package and among the different elements incentivized? What factors set the stage for successful widespread or nationwide adoption of financial incentives?
What unintended consequences and perverse incentives have been reported?
When can financial incentives be reduced or withdrawn entirely?
Do supply- and demand-side incentives work better if provided simultaneously, or is a mixed approach with multiple tools simply more costly for countries and donors?

## RESULTS AND DISCUSSION

### Overview of findings

The reviews and discussions of the Summit concluded that the use of FIs, particularly conditional cash transfers, increases utilization of maternal health services, which has been typically slow over the two decades since the Millennium Development Goals were established (10).

It is less apparent that such incentives improved the quality of maternal healthcare, primarily because of the lack of internationally agreed upon quality of care standards across the range of maternal health services. Metrics for measuring quality of care are needed both to determine the impact of incentive programmmes and what should be rewarded, particularly in the context of supply-side incentive programmes. The latter require indicators that can be routinely measured, collected, verified, and paid on, all without creating unintended negative consequences. Standardized quality of care criteria must be an immediate priority for maternal health experts.

The evidence of impact of the financial incentives on health outcomes is weak and of poor quality. However, impact measures, especially of significant change in the reduction of maternal mortality, are typically not feasible in the short timeframe and limited study populations described in the literature reviewed. Long-term studies should be undertaken to assess the sustainability and the health impact of FIs for maternal healthcare.

The Summit review also provided lessons for donors, researchers, programme implementers, and country-specific policy-makers to consider for future financial incentive programmes that are effective, ethical, and evidence-informed.

The specific findings of the evidence review on each of the supply- and demand-side mechanisms, including performance-based incentives, health insurance, user fee exemptions, conditional cash transfers, and vouchers, are presented in the papers of this Supplement. Morgan *et al*. (in this Supplement) summarizes these findings and recommendations across all incentive instruments on coverage, quality of maternal care, impact, implications for research, programme design, and implementation.

### Conclusions

Financial incentives for maternal health inherently involve both health and financial sectors. Therefore, it was not surprising that the Evidence Summit identified a lack of a strategic alignment between maternal health and economic expertise in the design, implementation, and assessment of financial incentive schemes for maternal health. Increased collaboration between the economic and maternal health communities is required to better inform the design of research to assess effectiveness and understand the ‘hows’ of programmes. Furthermore, maternal health experts and programme designers need to be more conscious of the implications for health systems of incentive programmes they support. A closer collaboration between finance and health sectors can enhance programmes and policy to deliver informative data and conclusions to governments of the low- and middle-income countries and donors on what the most sustainable, equitable, and effective incentive programmes are to improve maternal and neonatal survival.

Very few studies considered, evaluated, and reported on the effect of incentives on both supply and demand sides. Whether designed as supply- or demand-side programmes, it is useful to measure effects on both supply and demand sides.

There is also a need for prioritized research agenda to develop more evidence. This agendum needs to be developed among key partners, including governments, development banks, donors, and universities, with the balanced input of economists and public health specialists knowledgeable about maternal health. Duration of initiatives and appropriate timing of measurement of results need to be specified to ensure that research documents both short- and long-term impacts of financial incentives.

### Reason for hope

There is an urgent need for action, as we approach 2015, for evaluation of the progress towards MDG 5 and planning for the post-2015 world where ending the preventable maternal death needs to remain a priority (11), with an evidence-informed path to achieving this goal. While the current global rate of progress in maternal mortality reduction and the coverage of maternal health services are still below the level of progress needed to reach MDG 5 by 2015, there is a general consensus on the technical approach to improving survival during pregnancy, labour, delivery, and the immediate postpartum period. In addition, there has been a call for ensuring Universal Health Coverage (12), and financial incentives could become a major factor in making that vision become a reality. Linked with positive global trends in factors associated with reduced maternal risk, including reduced fertility rates, increased rates of female education, and increased per-capita gross domestic product, we have an unprecedented opportunity to accelerate progress. Creating demand rapidly for and improving the supply of quality maternal health services are challenges shared across many areas of global health but it is particularly compelling in maternal health, given the high mortality rates for mothers and their children who die near the time of birth or are left behind. Furthermore, there are often serious continuing consequences, physical and mental, for mothers and their newborns who survive with disabilities. When planned and used appropriately, financial incentives can be the effective means to improve the utilization and quality of maternal care to help achieve our goal.

## ACKNOWLEDGEMENTS

This work was carried out by US Federal Government Employees and persons supported through contractual mechanisms by the federal government. This is an open-access article under the terms of the Creative Attribution License which permits unrestricted use, distribution, and reproduction in any medium, provided that the original authors and source are credited. The views and opinions in this paper are those of the authors and not necessarily of the United States Agency for International Development nor the National Institute of Allergy and Infectious Diseases. Among the authors, MES is paid by the United States Agency for International Development. ESH is paid by the National Institute of Allergy and Infectious Disease and was on assignment with the Bureau for Global Health at the United States Agency for International Development during the planning of the Evidence Summit. MK is currently paid by the United States Agency for International Development through an Intergovernmental Personnel Act Assignment Agreement with Johns Hopkins University School of Public Health and, during the summit planning, was paid by John Snow, Inc.
